# Optimising a behavioural intervention to support endocrine therapy adherence for women with breast cancer: protocol for the ROSETA optimisation factorial randomised controlled trial

**DOI:** 10.1186/s13063-026-09765-6

**Published:** 2026-05-11

**Authors:** Sophie M. C. Green, Emma McNaught, Christopher D. Graham, Aaron Dowse, Hollie Wilkes, Pei Loo Ow, Elizabeth Travis, Robbie Foy, David P. French, Louise H. Hall, Rebecca Walwyn, Florence Day, Christopher Taylor, Andrew Carter, Daniel Howdon, Jane Clark, Jo Waller, Jacqueline Buxton, Sally J. L. Moore, Catherine Parbutt, Galina Velikova, Amanda Farrin, Michelle Collinson, Samuel G. Smith

**Affiliations:** 1 Leeds Institute of Health Science, University of Leeds, Leeds, LS2 9NL UK; 2 Clinical Trials Research Unit, Leeds Institute of Clinical Trials Research, University of Leeds, Leeds, LS2 9NL UK; 3 Department of Psychological Sciences and Health, University of Strathclyde, Glasgow, G1 1QE UK; 4 Division of Psychology and Mental Health, University of Manchester, Manchester, M13 9PL UK; 5 Department of Clinical and Health Psychology, Leeds Teaching Hospitals NHS Trust, Leeds, LS9 7TF UK; 6 Wolfson Institute of Population Health, Queen Mary University of London, London, E13 8SP UK; 7 Independent, Leeds, UK; 8 Medicines Management and Pharmacy Services, Leeds Teaching Hospitals NHS Trust, Leeds, LS9 7TF UK; 9 Leeds Institute of Medical Research, University of Leeds, Leeds, LS9 7TF UK

**Keywords:** Breast cancer, Medication adherence, Adjuvant endocrine therapy, Optimisation trial, Multiphase Optimisation Strategy, Factorial trial, Acceptance and commitment therapy, Text messaging, Screening trial

## Abstract

**Background:**

Adjuvant endocrine therapy (AET) reduces breast cancer recurrence and mortality. However, up to three quarters of women with breast cancer do not take AET as prescribed. Existing interventions to support adherence have shown limited effectiveness and often do not target the range of barriers to appropriate AET use. We developed four intervention components targeting barriers to AET adherence: Short Message Service (SMS) messages targeting forgetfulness, an information leaflet targeting medication beliefs, a self-management website targeting side-effects, and an acceptance and commitment therapy-based guided self-help programme targeting psychological flexibility. In the preparation phase of the Multiphase Optimisation Strategy (MOST), we conducted an external pilot optimisation trial. We met predefined progression criteria regarding consent, component adherence and availability of outcome measures, and concluded progression to an optimisation randomised controlled trial (O-RCT) was warranted. Our primary aim is to optimise the intervention package to support adherence to AET in women with early-stage breast cancer.

**Methods:**

We will conduct a multi-centre, individually randomised superiority O-RCT using a 2^4^ factorial design, with nested mixed-methods process and economic evaluations. We will randomise 512 women with early-stage breast cancer who have been prescribed AET to one of sixteen experimental conditions, operationalised as factors with two levels (on/off). Each condition is comprised of unique combinations of the intervention components. All participants will receive usual care. Our primary outcome is self-reported medication adherence at 12 months post-randomisation. Key secondary and process outcomes include quality of life, self-efficacy, habit formation, medication beliefs, psychological flexibility and distress, completed at 4, 8 and 12 months post-randomisation. Within the process evaluation, semi-structured interviews with participants will be conducted 5 and 13 months post-randomisation, and with trial therapists following intervention delivery. Cost per incremental quality-adjusted life year will be estimated in a health economic evaluation.

**Discussion:**

Within the optimisation phase of the MOST framework, this trial, using a complex factorial design, will enable us to build a more effective, affordable, scalable and efficient intervention package to support AET adherence in women with breast cancer. This approach will advance intervention science by simultaneously testing the mechanisms through which the intervention components are operating.

**Trial registration:**

International Standard Randomised Controlled Trial Number ISRCTN17334319. Registered on 02/02/2024.

**Supplementary Information:**

The online version contains supplementary material available at 10.1186/s13063-026-09765-6.

## Administrative information

**Table:** 

Title {1}	Optimising a behavioural intervention to support endocrine therapy adherence for women with breast cancer: protocol for the ROSETA optimisation randomised controlled trial
Trial registration {2a and 2b}	Registry: International Standard Randomised Controlled Trial Number ISRCTN: 17334319 The ROSETA Optimisation Trial – Investigating strategies to improve medication adherence in women with early-stage breast cancer https://www.isrctn.com/ISRCTN17334319 Date registered: 02/02/2024
Protocol version (1)	5.0, 28/01/2025
Funding {4}	This trial is independent research supported by the National Institute for Health and Social Care Research NIHR Advanced Fellowship, Professor Smith, NIHR300588. Professor Smith also acknowledges a Yorkshire Cancer Research University Academic Fellowship (L389SS). Dr Green acknowledges funding from an NIHR Development and Skills Enhancement Award (NIHR305132). Professor Foy acknowledges grant funding from the NIHR. Prof Walwyn acknowledges funding from an NIHR Advanced Fellowship (NIHR301709). Associate Professor Collinson reports NIHR, Yorkshire Cancer Research, Macmillan Cancer Support, Breast Cancer Now and British Lung Foundation grant funding paid to her institution, being a DMEC and TSC member of NIHR funded projects and being a member of NIHR grant funding panels. Professor Farrin reports NIHR grant funding paid to her institution, and reports being a DMEC and TSC member of NIHR and British Heart Foundation funded projects, a member of the global Ageing Research Trialists collaborative, and an NIHR Senior Investigator. The recruitment SWAT (Implement SWATs) is funded by NIHR Advanced Fellowship (NIHR302256). The views expressed in this protocol are those of the author(s) and not necessarily those of the NHS, the National Institute for Health and Social Care Research or the Department of Health and Social Care
Author details {5a}	1. Leeds Institute of Health Science, University of Leeds, Leeds, LS2 9NL 2. Clinical Trials Research Unit, Leeds Institute of Clinical Trials Research, University of Leeds, Leeds, LS2 9NL 3. Department of Psychological Sciences and Health, University of Strathclyde, Glasgow, G1 1QE 4. Division of Psychology and Mental Health, University of Manchester, Manchester, M13 9PL 5. Department of Clinical and Health Psychology, Leeds Teaching Hospitals NHS Trust, Leeds, LS9 7TF 6. Wolfson Institute of Population Health, Queen Mary University of London, London, E13 8SP, 7. Independent 8. Medicines Management and Pharmacy Services, Leeds Teaching Hospitals NHS Trust, Leeds, LS9 7TF 9. Leeds Institute of Medical Research, University of Leeds, Leeds, LS9 7TF
Name and contact information for the trial sponsor {5b}	University of Leeds, The Secretariat, Level 11 Worsley Building, University of Leeds, LS2 9JT. Tel: 0113 343 7587 Email: governance-ethics@leeds.ac.uk
Role of sponsor {5c}	The Sponsor is responsible for trial initiation management and financing of the trial as defined by Directive 2001/20/EC. These responsibilities are delegated to the CTRU as detailed in the trial contract. The Sponsor had no role in the design of this trial, nor will it have any role during its execution, analyses, interpretation of data or decision to submit results

## Introduction

### Background and rationale {6a}

Breast cancer is the most common cancer worldwide and is the fifth leading cause of cancer mortality [1]. Worldwide incidence of new cases is projected to increase by 38% by 2050 [2]. Around 80% of breast cancers are oestrogen receptor positive (ER+) [3]. Adjuvant endocrine therapy (AET), including selective serotonin receptor modulators (SERMs; e.g. tamoxifen) and aromatase inhibitors (AIs; e.g. anastrozole), is prescribed for 5–10 years to reduce the risk of recurrence and mortality in women with ER+ breast cancer [4–8]. However, up to three quarters of women are not adherent to AET [9–11]. Lower adherence is associated with increased risk of recurrence and mortality, reduced quality-adjusted life years, and increased healthcare costs [12–14]. 

There are several intentional and unintentional barriers to adherence to AET, including side-effects (e.g. hot flushes, joint pain, night sweats), psychological distress, concerns about AET that outweigh beliefs about its perceived benefits, and forgetfulness [15, 16]. Most interventions aiming to support AET adherence have not been effective, potentially because a limited number of barriers have been targeted to date, and there has been a tendency to rely on educational interventions [17–19]. A meta-analysis, including 25 unique studies, representing 367,873 women, found a small positive effect of interventions on AET adherence (odds ratio, 1.412; 95% CI, 1.183 to 1.682; *p* = 0.0001) [14]. However, the authors highlighted a lack of understanding regarding which components of the complex interventions are most effective, with two exceptions: side-effect management education, which was consistently ineffective, and reducing medication costs, which was consistently effective [15]. Typically, multicomponent interventions targeting AET adherence have been evaluated using a single-arm or parallel-group randomised trial (RCT). These designs are suitable for evaluating the effectiveness of the intervention package as a whole but cannot distinguish the effects of individual intervention components [20]. As such, we have limited understanding of which components of complex interventions are effective, limiting scientific progress [15].

The Multiphase Optimisation Strategy (MOST) is an engineering-inspired framework that aims to optimise multicomponent interventions to balance intervention effectiveness with affordability, scalability and efficiency [20, 21]. MOST advocates for an optimisation phase after intervention development and prior to definitive evaluation in which fully powered, efficient experimental designs can be used to empirically estimate the main and interaction effects of intervention components [20]. Data from an optimisation-randomised controlled trial (O-RCT) can be used to build an optimised intervention package that balances effectiveness with key implementation constraints such as cost or clinician time [20].

**Fig. 1:**
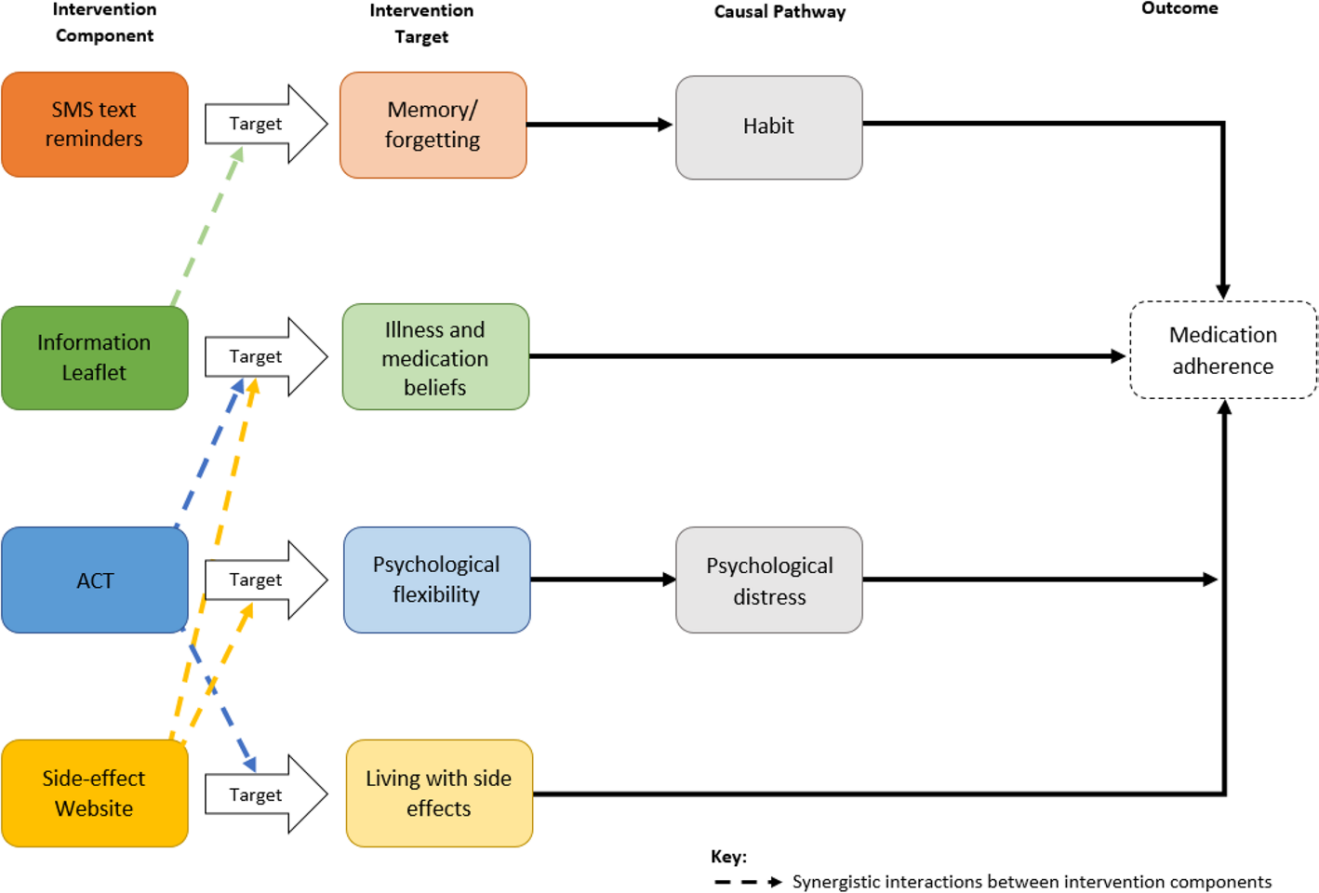
Conceptual model of ROSETA intervention. Figure taken from Green et al. [16]

As part of the ‘Refining and Optimising a behavioural intervention to Support Endocrine Therapy Adherence’ (ROSETA) programme, we used intervention mapping to develop a theory-based multicomponent intervention targeting four key barriers to AET: (1) SMS messages to target forgetfulness; (2) an information leaflet to target beliefs about the medication; (3) guided self-help based on acceptance and commitment therapy (ACT) to increase psychological flexibility and reduce psychological distress; and (4) a self-management website to support coping with side-effects (see Fig. 1 for conceptual model including hypothesised interactions) [16, 22, 23]. During the preparation phase of MOST, we conducted a multi-site external randomised pilot trial, using a 2^4–1^ fractional factorial design (ISRCTN10487576) [24, 25]. We randomised 52 women prescribed AET to one of eight conditions, each comprising a unique combination of the four intervention components. We met all predefined progression criteria for the consent rate, intervention component adherence and availability of outcome measures, which demonstrated an O-RCT was feasible and warranted. Our mixed-methods process evaluation suggested all intervention components were acceptable and could be delivered with adequate fidelity [26, 27]. Minor adaptations were made to the intervention components and trial processes prior to the O-RCT to further improve acceptability and fidelity [26, 27].

### Objectives {7}

This trial represents the optimisation phase of MOST (ISRCTN17334319). We aim to undertake an O-RCT to optimise the ROSETA intervention package. Our primary objective is to determine the most effective intervention package for supporting AET adherence at 12 months post-randomisation, using self-reported adherence data by estimating all main effects and interactions of each intervention component across all experimental conditions. As secondary objectives, we will also seek to optimise the intervention package using secondary outcomes (e.g. NHS prescribing and/or dispending data, global quality of life, and self-efficacy), and apply constraints on the optimal intervention, such as cost. We will estimate the main effects and interactions of our intervention components on key secondary outcomes outlined within our conceptual model. We will estimate the cost of delivering each intervention component. In the nested process evaluation, we will empirically test and refine our conceptual model, and monitor fidelity of training, delivery, receipt and enactment. Detailed objectives and endpoints are available in Additional file 1.

### Trial design {8(28)}

This is a multi-centre, individually randomised superiority O-RCT using a 2^4^ factorial design, with nested process and economic evaluations. Four candidate intervention components (SMS messages, information leaflet, ACT and website) will be operationalised as factors with two levels (on/off). There are 16 experimental conditions, made up of each combination of the factor levels (Table 1). All participants will receive usual care (UC) and up to four intervention components. There will be an equal chance of allocation to each of the 16 conditions. The trial adheres to the factorial extension of the Standard Protocol Items: Recommendations for Interventional Trials (Additional file 2) [29].

**Table 1:** ROSETA 2^4^ factorial design

Condition	Usual care	Text reminders	Information leaflet	ACT	Side-effect website	Estimated randomisation sample size
1	Yes	Yes	Yes	Yes	Yes	32
2	Yes	Yes	Yes	Yes	No	32
3	Yes	Yes	Yes	No	Yes	32
4	Yes	Yes	Yes	No	No	32
5	Yes	Yes	No	Yes	Yes	32
6	Yes	Yes	No	Yes	No	32
7	Yes	Yes	No	No	Yes	32
8	Yes	Yes	No	No	No	32
9	Yes	No	Yes	Yes	Yes	32
10	Yes	No	Yes	Yes	No	32
11	Yes	No	Yes	No	Yes	32
12	Yes	No	Yes	No	No	32
13	Yes	No	No	Yes	Yes	32
14	Yes	No	No	Yes	No	32
15	Yes	No	No	No	Yes	32
16	Yes	No	No	No	No	32

## Methods: participants, interventions and outcomes

### Study setting {9}

Trial sites will be secondary care NHS Trust hospitals providing services for breast cancer patients. Sites expressing an interest in the trial will be invited to complete a site feasibility questionnaire to verify that the team at the site is willing and able to comply with trial requirements to confirm capacity and capability. We will maintain a summary of participating sites and reasons for non-selection. Participating sites will be required to have obtained local ethical and management approvals and to undertake appropriate training in trial procedures (including a site initiation Q&A and, where relevant, the ACT intervention training) prior to the start of recruitment into the trial. A list of all participating trial sites is available at https://ctru.leeds.ac.uk/roseta/roseta-news/. The ACT component was delivered by therapists working in NHS sites, with two models of delivery whereby the therapist (1) treats patients from the same site the therapist is working from, or (2) treats patients at a different site than their own. 

### Eligibility criteria {10}

Eligible participants will be adult women with early-stage (1–3) breast cancer, prescribed AET (Table 2). Participants meeting the eligibility criteria will be eligible to receive all factors. Therapists eligible to deliver the ACT intervention component will fall into one of the following categories: Health and Care Professional Council (HCPC) registered practitioner psychologist (Clinical, Health or Counselling Psychologist), a UK Council for Psychotherapy (UKCP) registered psychotherapist, an assistant psychologist, a clinical associate psychologist or an individual with formal experience or training in delivering manualised psychological therapy for supporting well-being (e.g. a psychological well-being practitioner).

**Table 2:** Participant eligibility criteria

Inclusion	Exclusion
1. Capacity to provide informed consent 2. Women with early stage (1—3) breast cancer according to the Tumor, Node, Metastasis (TNM)/American Joint Committee on Cancer (AJCC) staging system^a^ 3. Aged ≥ 18 years 4. Have sufficient proficiency in English to be able to adhere to all intervention components and data collection required 5. Treated with curative intent 6. Completed their hospital-based treatment (e.g. surgery, radiotherapy and/or chemotherapy) for the current breast cancer within the last 24 months^b^﻿ 7. Currently prescribed oral AET (tamoxifen, raloxifene, anastrozole, letrozole, exemestane) 8. Access to a mobile phone to receive SMS messages* 9. Access to a computer or smart device that can access the internet*	1. Stopped taking AET if it is clinically contraindicated according to clinical recommendation 2. Involved in a similar research trial where medication adherence is a primary outcome*,** 3. Currently attending psychotherapy/psycho-oncology/psychology/counselling services, for any clinical reason* Note: Women who have been referred, but do not have an appointment booked within 5 months of the discussion about ROSETA are eligible. Women who have been referred and have an appointment booked within 5 months of the discussion about ROSETA are not eligible 4. Need for treatment for a severe mental health disorder or crisis, which is likely to interfere with participation (e.g. active psychosis, bipolar disorder, significant issues with addiction or self-harm or expressing active suicidal ideation with active plans and intent*) 5. Auditory problems that would prevent the patient from participating in a telephone or video call, or hearing audio clips* 6. Taken part in the ROSETA Pilot trial

^a^Women being treated for a second primary breast cancer or a breast cancer local recurrence are eligible for the trial, providing at least one of the cancers is being treated with AET, and they meet all eligibility criteria. Women with bilateral breast cancer are permitted, providing at least one breast is affected by hormone receptor-positive disease

^b^Women are still eligible for the trial if they are being treated with abemaciclib or monoclonal antibody-based therapy such as trastuzumab, kadcyla, pertuzumab, and phesgo; these medications do not have to be completed within the 24 months stipulated within this criterion

*Source data for these items will be either partially or completely patient self-report

**Consented to and the trial is still being delivered

### Who will take informed consent? {26a}

Informed consent will be sought from eligible patients who agree to participate. Consent may be taken over the phone, via video call or face-to-face by an authorised staff member (research nurse or delegate). For consent taken over the phone or video call, a telephone consent script will be used which covers all clauses of the consent form, which the research nurse will sign and date on the participant’s behalf. If face-to-face consent is taken, both the patient and research nurse will sign and date the consent form.

### Additional consent provisions for collection and use of participant data and biological specimens {26b}

Not applicable, no samples collected.

## Interventions

### Explanation for the choice of comparators {6b}

Due to the 2^4^ factorial design used, there will be no separate control group [20]. Each factor will have two levels (on/off). As the main effect of each factor is calculated by comparing the mean of conditions where the factor is set to ‘on’, with the mean of conditions where the factor is set to ‘off’, each factor has its own comparator group (i.e. the mean of the conditions whereby that factor is set to its lower [‘off’] level). Participants in all 16 conditions will continue to have access to UC, which will be the standard care offered to women at this stage of their treatment for breast cancer and is likely to differ by recruiting site. UC often includes an end-of-treatment summary meeting with a breast cancer nurse, including a holistic needs assessment and information about local services. Follow-up is patient-initiated, whereby most patients discharged are given contact details of a breast cancer nurse they can speak to if they have concerns. The content of treatment-as-usual programmes will be collected retrospectively at the end of the trial period from recruiting sites. Sites will also inform the CTRU should any new guidance or support relating to encouraging medication adherence be implemented at sites during the trial.

### Intervention description {11a}

We used intervention mapping to develop four intervention components addressing barriers to AET adherence. The intervention components are described using the TIDieR checklist [30] (Additional file 3).

#### SMS Intervention Component (target: memory)

The SMS messages are based on behaviour change techniques hypothesised to support habit formation, and were co-developed by experts in behaviour change and women with breast cancer [23]. A series of 43 SMS messages will be delivered to participants randomised to receive the SMS component, to the mobile number they provided at registration. The SMS messages will be sent via a CTRU-developed automated system, delivered over a period of four months in varying frequency: 3 opening messages delivered up to 7 days post-randomisation, followed by daily messages for 2 weeks, 2 messages per week for 8 weeks, weekly messages for 6 weeks and a closing message after 4 months. Three messages (one per month) will inform participants that they can stop the SMS messages being sent. Participants can select the time of day the messages are sent from three options: 8am, 12 pm or 8 pm. All participants will receive the same messages in the same order.

#### Information leaflet intervention component (target: medication beliefs)

A 6-page information leaflet, previously optimised to support beliefs about medication necessity and reduce concerns in women taking AET, will be sent by the research nurse to participants via email, 1-week post-randomisation. The leaflet contains explanations of how AET works, detailed information about the benefits of AET, presence or absence of side-effects for each type of AET, answers to common concerns, and quotes from other women taking AET about their motivations and experiences [22]. The content of the leaflet is based on beliefs previously described in quantitative and qualitative research, views from our patient and public involvement group, the necessity concerns framework [31] and the common sense model of self-regulation [32].

#### Self-management website intervention component (target: side-effects)

Participants randomised to receive the website component will be sent log-in details by the research nurse one-week post-randomisation. The website contains information on managing side-effects, patient stories, and signposting for further information/places of support. The content of the website is informed by an umbrella review undertaken regarding strategies to self-manage AET side-effects [33].

#### Acceptance and commitment therapy intervention component (target: psychological flexibility and distress)

Acceptance and Commitment therapy aims to improve psychological flexibility [34, 35]. The ACT component is a guided self-help programme consisting of four modules which teach skills engendering different aspects of psychological flexibility: mindfulness, unhooking, following values and living beyond labels. Each module consists of a participant manual, audio files and home practice exercises. The modules are supported by five 25-min sessions with a therapist, which will take place via videoconferencing or telephone. In telephone sessions therapists aim to reinforce learning from skills practice, deepen connection with values and model psychological flexibility. Participants will be added to an ACT waiting list at their respective site with sessions beginning when a therapist is available. The time to receipt of the first ACT session will be monitored. All five ACT sessions should be delivered within 3 months of the first session. Within the support sessions, the therapist will discuss the module completed, experiences of home practice exercises, problem solve any difficulties and introduce the following module.

### Criteria for discontinuing or modifying allocated interventions {11b}

Participants are free to withdraw consent and leave the trial at any time, without giving reasons and without it affecting their care. Patients can withdraw from the SMS and ACT components, from questionnaire completion, and/or from collection of data from NHS England. Data collected up to the date of withdrawal will be used in analysis. Withdrawals will be recorded together with the reason for withdrawal where provided. A clinician within the clinical care team may decide a participant should be withdrawn if there is reason to believe they have become unsuitable for the trial. This may include but is not limited to suicidal ideation with active suicidal behaviours/plans and imminent intent, indication that the intervention components are leading to worse mental health, loss of capacity to consent, significant issues with drug or alcohol addiction, or clinical recommendation for the participant to stop taking AET. No modifications will be made to the intervention components.

### Strategies to improve adherence to interventions {11c}

Appointment reminders for the ACT component will follow usual practice at each site.

### Relevant concomitant care permitted or prohibited during the trial {11d}

Participants in all conditions can continue to access usual care during the trial. Participants who are currently attending psychological therapy for any clinical reason (e.g. for depression/anxiety) will be ineligible to take part in the trial (Table 2).

### Provisions for post-trial care {30}

There are no plans to compensate individuals who suffer harm from trial participation. Brief information will be provided within the participant information sheet and at the point of questionnaire completion to signpost participants to services in case they experience unexpected psychological distress. The Sponsor has cover for liabilities/prospective liabilities arising from negligent harm and in some circumstances, non-negligent harm. Clinical negligence indemnification rests with participating Trusts.

### Outcomes {12}

Assessments with associated timings are described in Table 3. All self-reported outcomes are detailed below. Endpoints for each objective are described in Additional file 1.

**Table 3:** Timeline of interventions and assessments

Assessment/intervention	Who is collecting/completing?	Timeline (months post-randomisation)				
		Baseline	4 months	8 months	12 months^a^	End of trial
Intervention components						
SMS messages	Participant	*Randomisation to 4-months*				
Information leaflet	Participant	*Delivered 1 week post-randomisation, can be used throughout the trial*				
Self-management website	Participant	*Access from one week post-randomisation to 12 months*				
Guided self-help ACT	Participant	*Delivered after randomisation, within a period of 3 months from first session*^*b*^				
Eligibility and consent						
Eligibility assessment of inclusion and exclusion criteria	Research nurse/delegate	X				
Consent	Research nurse/delegate	X				
Baseline and follow-up data						
Contact details	Research nurse/delegate	X	*CTRU to be notified of any changes*			
General demographics	Participant	X				
Education and employment	Participant	X				
Medical details and history	Participant/Research nurse/delegate	X				
DOSE-Nonadherence	Participant	X	X	X	X	
Prescribing/Dispensing Data (NHS England)^c^	Trial team				X	
EQ-5D-5L	Participant	X	X	X	X	
MQoL-R	Participant	X	X	X	X	
SEAMS	Participant	X	X	X	X	
SRBAI	Participant	X	X	X	X	
BMQ-AET	Participant	X	X	X	X	
MPFI	Participant	X	X	X	X	
DASS-21	Participant	X	X	X	X	
EORTC QLQ-C30	Participant	X	X	X	X	
EORTC QLQ-BR45	Participant	X	X	X	X	
EORTC IL133	Participant	X	X	X	X	
Intervention costs (NHS Reference Costs/PSSRU)	Trial team				X	
UK Cancer Costs	Participant		X	X	X	
AET Use	Participant	X	X	X	X	
Adherence to intervention components^d^	Participant		X			
Adherence to ACT	Participant			X		
Use of ACT skills	Participant		X	X	X	
Reconstructive surgery	Participant				X	
Status check	Research nurse/delegate		X			
Safety reporting	Research nurse/delegate		*Ongoing*			
Intervention data						
Intervention adherence data	Research nurse/delegate/trial team		*Ongoing*			
Trial therapist data						
Trial therapist demographics	Therapist	X				
Trial therapist training details	Therapist	X				
Procedural Fidelity checklist	Therapist		*Completed at the end of each ACT session*			
ACT-FM competence assessment (therapist stance subscale)	ACT intervention lead or delegate (A)/independent reviewer (I)		*Completed following ACT training (A)* *Completed after first 5 delivered sessions (A)* *Completed at the end of the trial on 20 sessions (I)*			
Usual care						
Usual care services offered at research site	Research nurse/delegate	X				X
Services accessed by participant	Research nurse/delegate					X
Process evaluation sub-study						
Consent	Researcher	X				
Interview with consenting participant	Researcher		*At 5 and 13 months*			
Interview with consenting therapist	Researcher		*One month prior to the end of intervention delivery*			

^a^Participants recruited during the final 4 months of recruitment will have reduced follow-up of between 8 and 11 months. 12-month data will therefore be collected early for participants meeting this criterion

^b^Participants will be added to an ACT waiting list at their respective site with sessions beginning as soon as a therapist is available. The time to receipt of the first ACT session will be monitored

^c^We will explore the possibility of using NHS England data to conduct longer-term follow-up (i.e. post 12-months randomisation) of those participants recruited early to the trial

^d^Receipt of intervention components, adherence to text reminders, adherence to information leaflet, adherence to website

#### Primary outcome

The Domains of Subjective Extent (DOSE)-Nonadherence 3-item self-report measure will assess the extent of non-adherence to AET over the past 7 days [36].

#### Secondary outcomes

EuroQol-5 Dimension 5 Level (EQ-5D-5L): a 5-item self-report health-related quality-of-life measure, assessing QoL over five components including mobility, self-care, usual activities, pain/discomfort and anxiety/depression [37].

McGill Quality of Life-Revised (MQoL-R): a 14-item self-report measure assessing physical well-being, physical symptoms, psychological symptoms, existential well-being and support, and overall quality of life [38].

Self-Efficacy for Appropriate Medication Use Scale (SEAMS): a modified, 6-item version of the SEAMS self-report measure will be used to assess self-efficacy for medication taking [39].

Self-Report Behavioural Automaticity Index (SRBAI): a 4-item self-report measure assessing habitual behaviour patterns [40].

Beliefs about Medicine Questionnaire (BMQ-AET): a 10-item self-report measure assessing specific medication beliefs on concern and necessity belief scales [41].

Multi-Dimensional Psychological Flexibility Index-Short version (MPFI): a 24-item self-report measure assessing psychological flexibility and inflexibility [42].

Depression Anxiety Stress Scales (DASS-21): a 21-item self-report measure comprising three subscales assessing depression, anxiety and distress [43].

European Organisation for Research and Treatment of Cancer (EORTC) QLQ-C30: a 30-item self-report measure assessing health-related quality of life in cancer patients [44].

EORTC QLQ-BR45: a 45-item self-report measure assessing QoL in patients with breast cancer [45].

EORTC-IL133: a 2-item self-reported measure created from the EORTC item library (https://qol.eortc.org/item-library/), to assess vaginal discharge and abnormal vaginal bleeding.

UK Cancer Costs Questionnaire (UKCC): an adapted version of the UKCC self-report measure to assess services and medications participants may have used while participating in the trial [46].

Adjuvant Endocrine Therapy Use: a 3-item self-report measure, created for the purpose of this trial, asking about AET prescribed, the date of first prescription, previous AET and any reasons for switching at baseline. At follow-up, 3 items will ask about any changes to the AET prescription in the previous four months and reasons for switching AET.

Adherence to Intervention Components: a self-report measure assessing adherence to the intervention components, created for the purpose of this trial. The measure comprises two items for each intervention component they were allocated to receive; asking whether they received the component and how much of the component they used. Participants randomised to the ACT component will be asked two additional questions about how much of the home practice tasks they completed, and how many of the audio files they listened to.

Use of ACT skills: a 4-item self-report measure, created for the purpose of this trial, assesses engagement with ACT skills.

Reconstructive Surgery: a 3-item self-report measure, created for the purpose of this trial, asking about reconstructive surgery, whether breast cancer medication was stopped before or after the therapy, and for how long.

### Participant timeline {13}

Table 3 describes the timeline for the interventions and all trial assessments.

### Sample size {14}

The trial is powered to detect small to moderate (*d* = 0.28) main effects for each intervention component on medication adherence, based on findings from a meta-analysis of 8 AET adherence trials [17]. This is with the intention that the optimised intervention package would result in a moderate to large effect (*d* = 0.50). This would be clinically meaningful with regard to mortality outcomes [13, 14, 47]. We estimate that 512 participants (*n* = 32 per condition) will provide 80% power to detect a main effect of 0.28 assuming a 5% significance level and 20% loss to follow-up. Treatment-related clustering due to ACT therapists was not accounted for in the sample size calculation. Our use of effect coding (− 1, + 1) means that all interactions will also be powered to the same level, assuming the same size of effect. As each comparison corresponds to a distinct hypothesis, no adjustment in significance level will be made for multiplicity. This aligns with the purpose of the trial to optimise a complex intervention rather than definitively evaluate it.

### Recruitment {15}

Participants will be identified and recruited from UK NHS hospital sites providing services for breast cancer patients. Sites who are recruiting or who are planning to recruit to other trials where the primary aim is to support adherence to AET will be reviewed to determine whether co-enrolment would be feasible in terms of recruitment to target and site capacity. Participants will be identified via four recruitment routes. In Route 1 a research nurse will screen patient records for potentially eligible patients, who will be imminently attending an upcoming appointment. In Route 2, patients who self-refer to their oncologist to discuss problematic medication side-effects and/or adherence within 24 months of completing their hospital treatment will be identified. In Route 3 a research nurse will retrospectively screen patient records for potentially eligible patients who have completed their hospital treatment within the last 24 months. The research nurse will post an invitation letter and information sheet to potentially eligible participants. Route 4 involves the self-referral of potential participants, identified via local breast cancer support groups and charities, targeted social media campaigns, and the Be Part of Research Volunteer Service. Interested participants will be directed to a trial website which contains all trial information and an initial screening questionnaire, which will be sent to the CTRU trial team. If the patient is likely to be eligible based on the initial details provided, the CTRU trial team will send the patient’s details to the relevant hospital site and will make the patient aware. The research nurse will contact potentially eligible participants via telephone. Participating sites will complete a screening form for all identified patients, including anonymised data regarding age, ethnicity, staging, tumour type and whether a patient is randomised. Reasons for non-randomisation will also be captured. For all routes, the research nurse will confirm eligibility and record informed consent, either remotely or in person.

A Study Within A Trial (SWAT) will be embedded to understand the effectiveness and cost-effectiveness of a charitable donation incentive for increasing participant recruitment rates. This recruitment SWAT, sponsored by the University of York, will evaluate the effectiveness of a £10 charitable donation, on an unconditional basis, versus no charitable donation (i.e. the usual invitation) on participant recruitment rates.

## Assignment of interventions: allocation

### Sequence generation {16a}

Participants will be randomised in an equal allocation ratio to one of 16 experimental conditions, ensuring randomisation to relevant levels of factors occurs at the same time point. Randomisation lists will be prepared by the CTRU statistical team using stratified permuted blocks with fixed block size, to ensure experimental conditions will be well balanced for recruitment route (route 1 vs. route 2 vs. route 3 vs. route 4) and age (< 50 and ≥ 50).

### Concealment mechanism {16b}

Randomisation will be performed using the online CTRU-automated 24-h randomisation service which ensures allocation concealment. The service is accessible to trained researchers with an authorisation code and pin.

### Implementation {16c}

Following confirmation of eligibility and informed consent, participants will be registered onto the trial by an authorised member of staff at the research site, using the CTRU automated 24-h registration service. Following registration, the participant will be emailed a link to complete the online baseline questionnaire, and the research nurse will collect baseline data from the participant’s medical records. Once the baseline questionnaire and medical record review have been completed, participants will be randomised by an authorised member of staff at the research site using the CTRU automated 24-h randomisation service.

Following successful randomisation, the person performing the randomisation, the principal investigator, and the relevant local research team at that research site will receive an automated email confirmation of randomisation, highlighting subsequent tasks required for the site i.e. notifying the participant of their allocation with an intervention summary document, and informing the participant’s General Practitioner (GP) of their participation via letter. For participants allocated to the ACT component, a nominated person at the corresponding psychology service will also receive the email notification highlighting that an appointment should be scheduled. For participants allocated to the website or information leaflet components, the research nurse will provide the website link and log-in details and/or the information leaflet one-week post-randomisation via email. The commencement of the SMS component will be coordinated by the CTRU.

## Assignment of interventions: blinding

### Who will be blinded {17a}

Participants, therapists, the research nurse or delegate who performs the recruitment process and the CTRU statistical team will not be blinded to allocation. It is not possible to blind the research nurse or delegate as they inform the participant of their randomisation allocation and coordinate some of the interventions. GPs will be informed about trial participation but will not be informed of a participant’s allocation. Clinical teams will not routinely be informed about randomisation allocation.

The trial design incorporates measures to reduce bias. Follow-up measures will be completed by participants online. Site staff can call participants to aid outcome measure completion and, wherever possible, a member of staff who is blind to the randomisation allocation will conduct these calls. If site staff are subsequently unblinded, subsequent data collection will be completed by an alternative member of the site team who is blind to allocation.

### Procedure for unblinding if needed {17b}

There is no procedure for unblinding, as participants, therapists and the research nurse or delegate performing the randomisation will not be blinded to allocation.

## Data collection and management

### Plans for assessment and collection of outcomes {18a}

#### Baseline assessments

For participants who are eligible and who provide written informed consent we will collect data at baseline and prior to randomisation including mobile phone number, email address, date of birth, postcode, gender, NHS number, marital status, ethnicity, number of children, menopausal status, employment status, education level, co-morbidities, year and stage of diagnosis, tumour type, breast cancer treatment received, endocrine therapy, supportive therapies and previous exposure to ACT/cognitive behavioural therapy or mindfulness. Self-reported participant outcomes described in detail in the outcomes section and Table 3 will also be collected from participants via Research Electronic Data Capture (REDCap).

#### Follow-up assessments

Follow-up assessments will be completed at 4-, 8- and 12-month post-randomisation (Table 3). This includes self-reported participant outcomes, cost data regarding the delivery of the ACT component, and prescribing/dispensing data from NHS England. We will explore the possibility of using NHS England data to conduct longer-term follow-up (i.e. post-12-month randomisation) for participants recruited early to the trial. Participants recruited during the final 4 months of recruitment will have reduced follow-up of between 8 and 11 months. This is to maximise the recruitment period, while adhering to funding constraints. The research nurse will perform a status check for each participant at 4, 8, and 12-month post-randomisation.

#### Intervention data

Intervention data regarding delivery of the website login details and information leaflet, SMS delivery and receipt, opt-out of SMS messages, website usage and ACT session summary details will be collected (Table 3).

#### Trial therapist data

Demographic data for all trained therapists will be collected including age, gender, qualifications and experience. Training details will record the date of training, deliverer, number of therapists in the session, type (initial/booster) and length of training.

Therapists will be asked to complete a procedural fidelity checklist at the end of each session, and to record attendance at the session and the number of appointments missed. This checklist will assess fidelity of delivery of the ACT component. Therapists will confirm whether they undertook key intervention procedures (e.g. reflection on the skills exercises, emailing next module content). The checklist includes 8 items for sessions 1 and 2, 7 items for session 3, 6 items for session 4 and 4 items for session 5. Therapists will additionally be asked to assess to what extent the participant engaged with the module materials.

An external reviewer will assess 20 randomly selected ACT sessions from sessions 2, 3, or 4 using the ACT-FM therapist stance subscale [48]. This measure assesses therapists’ fidelity to ACT principles when delivering a session. Four items assess ACT-consistent behaviours, and three items assess ACT-inconsistent behaviours. 

#### Usual Care

Sites will provide information on usual care during site set-up and at the end of the trial. Participants will be asked to self-report services accessed, including hospitalisations and mental-health referrals at baseline and follow-up. This information will also be collected by the research nurse at the end of the trial at a participant level.

### Plans to promote participant retention and complete follow-up {18b}

Email and/or telephone reminders will be used to encourage completion of follow-up questionnaires. Participants will receive a maximum of 2 reminders at each time point. Follow-up data collection over the phone by the research nurse is permitted. A retention SWAT will be embedded to understand the effect of SMS pre-notifications and reminders on questionnaire return, which will include additional contact via SMS at each follow-up time point [49, 50].

### Data management {19}

Data will be monitored for completeness by the CTRU trial team, using established verification, validation and checking processes. For the recruitment SWAT, a collaboration agreement will be in place between the University of Leeds and the University of York outlining the data requirements.

### Confidentiality {27}

All information collected during the trial will be kept strictly confidential on paper and electronically at the CTRU and Leeds Institute of Health Sciences (LIHS) as appropriate. All aspects of the Data Protection Act 2018 will be followed. At the end of the trial, all data held by the CTRU and all trial data will then be securely archived at the University of Leeds in line with the Sponsor’s procedures for a minimum of 5 years.

### Plans for collection, laboratory evaluation and storage of biological specimens for genetic or molecular analysis in this trial/future use {33}

Not applicable, no samples collected.

## Nested process evaluation

A mixed-methods approach will involve trial participants and trial therapists delivering the ACT component. Quantitative assessments will use participant questionnaires and trial data (Table 3). Optional semi-structured interviews with trial participants will take place at 4–5 months and 12–13 months post-randomisation. Interviews with participants will focus on mechanisms of effect, acceptability, fidelity of receipt and enactment of the intervention components. Maximum purposive sampling will be used to interview participants from a range of ages across all four intervention components. We will oversample at the first time point (*n *= 40) to account for approximately 50% attrition at the subsequent interview (*n* = 20), to ensure the sample holds sufficient information power [51].

Interviews with trial therapists (*n* > 5 until sufficient information power) will take place at the end of the intervention delivery period (or at the point of therapist withdrawal), and will focus on the adequacy of training, the practicality of delivering the ACT component, suggested improvements, barriers and enablers to training and delivery of the ACT component, and acceptability of the ACT component. Purposive sampling will be used to prioritise assistant psychologists, clinical associate psychologists and psychological well-being practitioners, as higher bands of therapists have already been interviewed in the pilot trial. Trial participants and trial therapists will provide informed verbal consent for the interviews. All interviews will be conducted via telephone or videoconferencing software and transcribed verbatim using in-built auto transcription or by an external company with a data sharing agreement.

## Health economic evaluation

The economic evaluation will adopt the NICE reference case employing a primary outcome of cost per incremental quality-adjusted life year (QALY). Costs will include the costs of the interventions and healthcare resources used in primary and secondary care, based on patient self-report and data obtained from hospital records. Unit costs will be taken from publicly available datasets from the relevant PSSRU report on Unit Costs of Health, NHS Reference Costs, and the estimate of costs of prescribed medication relevant to the setting (NHS Drug Tariff, Electronic Market Information Tool (eMIT), and the British National Formulary). Health outcomes will be assessed by the EQ-5D-5L and converted to QALYs using the relevant value set.

## Statistical methods

### Statistical methods for primary and secondary outcomes {20a}

#### Primary endpoint analysis

The DOSE-Nonadherence extent scale will be scored as per author guidance and summarised overall and by factor at baseline and 12 months post-randomisation using descriptive statistics. The primary analysis will use a linear regression model. The four main effects, the eleven interactions (all two-way, three-way and four-way) together with the stratification factors (recruitment route and age group) and the DOSE-Nonadherence score at baseline will be included as independent variables in a single analysis model. This approach is pre-specified and will not be based on estimated interactions. Model fit will be explored and poorly fitting models may lead to additional sensitivity analyses.

Effect coding (− 1, + 1) will be used for every factor in the analysis model to ensure the estimation of main effects rather than simple effects as would be obtained via dummy coding (0, + 1); that is, the average effect of each factor across all levels of the other factors, rather than the effect of a component at a particular level of the other factors.

Type III sums of squares, mean square, and *p*-values will be presented for overall effects; as will the parameter estimates, associated standard error and *p*-value for each covariate. Due to the nature of effect coding, parameter estimates represent ½ the main effect and ½ the interaction effects. Graphical plots (e.g. Pareto plot) will be presented to further explore the main and interaction effects of the intervention components. Participants recruited during the final 4 months of recruitment will have a shorter duration of follow-up (8–11 months). Data from the final planned follow-up for these participants will be included in this analysis.

#### Secondary endpoint analysis

Secondary participant-reported outcomes (DOSE-Nonadherence, EQ-5D-5L, MQoL-R, SEAMS) will be scored as per author guidance and summarised overall and by intervention component at baseline, 4-, 8- and 12-months post-randomisation using descriptive statistics. These endpoints will be analysed in the same manner as the primary endpoint with the outcome in question at baseline replacing the DOSE-Nonadherence score at baseline in the model. Data from the final planned follow-up for these participants will be included in the analysis at 12-months post-randomisation. Missing data will also be explored and incorporated as per the primary analysis.

Prior to the development of the statistical analysis plan, we will explore the potential to obtain NHS prescribing and/or dispending data and determine the most appropriate analysis strategy. This may include but is not restricted to time to medication cessation (i.e. time-to-event), proportion of days covered (i.e. continuous) or proportion adherent (i.e. binary). The proposed modelling approach for this outcome will be fully specified in the statistical analysis plan before any analysis is undertaken.

SRBAI (SMS component), BMQ-AET (information leaflet component), MPFI (ACT component), DASS-21 (ACT component), EORTC QLQ-C30 (website component), EORTC QLQ BR-45 (website component) and EORTC IL133 (website component) will be scored as per author guidance and summarised overall and by intervention component at baseline, 4-, 8- and 12-months post-randomisation using descriptive statistics. These endpoints will be analysed in the same manner as the primary endpoint with the outcome in question at baseline replacing the DOSE-Nonadherence score at baseline in the model.

## Decision-making

A decision-making phase following an O-RCT is essential [52]. The O-RCT data will be used to decide which levels of which intervention components will make up the optimised intervention package. We will take a decision-priority perspective, following the component screening approach recommended for use in the MOST framework [52]. For our primary aim, we will use the all-active components criterion, which is defined as the best expected outcome irrespective of constraints such as cost.

A component will be considered ‘important’ if it has a main effect or interaction effect meeting *p* < 0.05. Main and interaction effects meeting this threshold will be added to the parsimonious prediction model, and all other coefficients will be set to zero. We will first screen in any intervention components with a positive main effect on the outcome and will screen out any intervention components with no effect, or a negative effect on adherence. We will then reconsider the screened-in and screened-out lists based on important interactions. Lower-order interactions, interactions containing a component where a main effect was present, and interactions hypothesised in our conceptual model (Fig. 1) will be prioritised [53]. Interaction plots will be examined to aid decision-making. Components in the screened-in list at the end of this process will be set to their higher levels, and components in the screened-out list will be set to their lower level. This will form the optimised intervention package.

### Interim analyses {21b}

No interim analysis is planned. Blinded interim reports will be presented to the trial management group (TMG) and trial steering committee (TSC) containing descriptive information relating to recruitment, follow-up, safety and data quality.

### Methods for additional analyses (e.g. subgroup analyses) {20b}

Any subgroup analysis will be described in the statistical analysis plan, prepared prior to any analysis being undertaken.

### Methods in analysis to handle protocol non-adherence and any statistical methods to handle missing data {20c}

A per-protocol analysis of the primary outcome will also be conducted based upon predefined criteria associated with intervention adherence. Adherence to each intervention component is defined as receiving 75% of SMS messages with no opt-out, reading ‘at least some of’ the information leaflet, completing 2/4 ACT modules, and registering and logging onto the website at least once. For primary and secondary analyses, we will explore missing data patterns and reasons for missingness to guide our assumptions around missing data. If the data are missing completely at random, complete-case analysis will be used. If the data are not considered to be missing completely at random then imputation will be used. The imputation strategy will be defined in full in a predefined statistical analysis plan prior to analysis commencing.

### Plans to give access to the full protocol, participant-level data and statistical code {31c}

To maintain the scientific integrity of the trial, data will not be released prior to the end of the trial, either for trial publication or oral presentation purposes, without the permission of the TSC, the chief investigator, and the CTRU. Requests to access trial data should be made to ctru-dataaccess@leeds.ac.uk. Requests will be reviewed by relevant stakeholders. No data will be released before an appropriate agreement is in place setting out the condition of release. An open-access data set containing anonymised data will be prepared to allow other researchers to carry out further research in the public interest.

### Process evaluation analysis

A full statistical analysis plan will be generated prior to analysis taking place. All validated participant-reported outcomes will be scored as per author guidance. Structural equation modelling will be used to empirically test the conceptual model [54]. Latent transition analysis will be used to understand whether there are latent trajectories of the mediating variables and what demographic and clinical characteristics are associated with class membership [55, 56]. Thematic analysis and ideal types analysis will be used to analyse the qualitative data cross-sectionally and longitudinally [57]. Frequencies and descriptive statistics will be used to summarise the quantitative fidelity assessments. A triangulation protocol will be used to assess agreement, partial agreement, silence or dissonance between the qualitative and quantitative findings from objectives 1–4 [58].

### Health economic analysis

A within-trial analysis, adopting a health and social care perspective, will be conducted to estimate impacts on both costs and health-related quality of life. This will allow an appropriate summary measure of cost-effectiveness—net health benefit—to be calculated, and uncertainty around these estimates to be characterised. These results will also be presented as incremental cost-effectiveness ratios with clear guidance given on their interpretation. Uncertainty around these summary measures will be presented using a cost-effectiveness acceptability curve, populated by the output of the seemingly unrelated regression. These health economic outcomes will be calculated from data collected at 4, 8 and 12 months (or equivalent).

## Oversight and monitoring

### Composition of the coordinating centre and trial steering committee {5d}

Three groups will oversee the trial. The TSC will have overall responsibility for the oversight of the trial, including trial progress, adherence to protocol, participant safety and consideration of new information. The TSC will include an independent chair, at least two other independent members and a PPI representative. The TSC will meet at least annually. The TMG will comprise the chief investigator, co-investigators, CTRU delivery team, other key external members of staff involved in the trial, and a consumer representative. The TMG will have responsibility for the set-up, ongoing management, promotion of the trial and for the interpretation and publishing of the results. The TMG will meet at least quarterly. The user advisory group will be comprised of four patient representatives, chaired by a member of the trial team. They will review trial materials to advise on the suitability of the content, methods and materials, review and advise on dissemination materials, and will contribute to discussions on patient recruitment, consent and any other relevant topics. They will meet approximately twice yearly throughout the trial period.

### Composition of the data monitoring committee, its role and reporting structure {21a}

As the level of risk involved is low with regard to patient safety, there will be no separate Data Monitoring and Ethics Committee convened. The TSC will adopt a safety monitoring role, with the constitution of a sub-committee to review safety issues where this becomes necessary.

### Adverse event reporting and harms {22}

In this population, we expect that episodes of acute illness, infection, new medical problems and deterioration of existing medical problems will occur and could result in prolonged hospitalisation, hospital re-admission, significant or permanent disability or incapacity, or death. In recognition of this, events fulfilling the definition of a serious adverse event (SAE) will not be subject to expedited reporting in this trial, unless the event results from administration of any research procedure and fulfils the definition of a related or unexpected serious adverse event (RUSAE). Additionally, we might expect to see low mood (including suicidal thoughts and plans), fatigue, anxiety and/or psychological distress. In recognition of this, any events relating to these factors will not be reported as a RUSAE. As deaths and hospitalisations are expected within the trial population, they will not be subject to expedited reporting to the REC, unless the TSC advises that the frequency of deaths or hospitalisations observed within the trial population is significantly higher than that expected in this population.

Reports of new episodes of physical self-harm in people who have not previously self-harmed will be considered SAEs and will be assessed for relatedness and unexpectedness. We do not expect participants to report an intention to carry out any reported thoughts and plans regarding suicide. Therefore, if this is identified, it will be considered an SAE and will be assessed for relatedness and unexpectedness. We will also monitor the proportion of participants who report ‘extreme’ scores on the DASS at follow-up, who did not have ‘extreme’ scores at baseline, following the scoring definitions in the DASS scoring manual. The number of participants meeting these criteria will be routinely reported to the TMG and TSC.

All reportable events will be reviewed by the chief investigator and those meeting the definition of an RUSAE will be subject to expedited reporting to the Sponsor and the main REC by the CTRU on behalf of the chief investigator within 15 days.

### Frequency and plans for auditing trial conduct {23}

Protocol compliance will be assessed throughout the trial. Protocol deviations, unplanned non-compliance or breaches are considered departures from the approved protocol. These processes will not be independent from investigators and the sponsor.

### Plans for communicating important protocol amendments to relevant parties (e.g. trial participants, ethical committees) {25}

Amendments are submitted for review to the research ethics committee following agreement of the changes by the central trial team, chief investigator, and the Sponsor. Upon approval, the central trial team is responsible for communication of the changes to all participating site teams and provision of updated documents/procedures. Confirmation of implementation of the changes associated with the amendment is tracked and logged to ensure adherence. Should any changes result in the requirement of reconfirmation of consent, the site research teams will have the responsibility of contacting participants, sharing the updated information, and gaining consent.

### Dissemination plans {31a}

Results will be presented at scientific meetings and published in international peer-reviewed journals. Authorship decisions will be guided by the International Committee of Medical Journal Editors criteria. Summaries will be provided to participants and the trial funder.

## Discussion

This paper describes the design and methods of the ROSETA O-RCT, which aims to optimise an intervention package to support adherence to AET in women with early-stage breast cancer. The novel design will advance our understanding as to which intervention components are effective in supporting adherence to AET. Once the optimised intervention package has been established, we anticipate proceeding to the evaluation phase of the MOST framework, in which a definitive evaluation of the intervention will be undertaken.

## Trial status

Recruitment for this trial commenced in May 2024 and is anticipated to conclude by May 2026. Protocol version 5.0, 28/01/2025.

## Supplementary Information

Additional file 1. Objectives and Endpoints: detailed objectives with linked endpoints.

Additional file 2. SPIRIT Factorial trial extension checklist.

Additional file 3. TIDieR checklist of intervention components.

## Data Availability

To maintain the scientific integrity of the trial, data will not be released prior to the end of the trial, either for trial publication or oral presentation purposes, without the permission of the TSC, the CI, and the CTRU. Requests to access trial data should be made to ctru-dataaccess@leeds.ac.uk. Requests will be reviewed by relevant stakeholders. No data will be released before an appropriate agreement is in place setting out the condition of release.

## Abbreviations

ROSETA: Refining and Optimising a behavioural intervention to Support Endocrine Therapy Adherence
AET: Adjuvant endocrine therapy
SMS: Short Message Service
O-RCT: Optimisation randomised controlled trial
MOST: Multiphase Optimisation Strategy
UC: Usual care
ER+: Oestrogen receptor positive
SERMs: Selective oestrogen receptor modulator
AI: Aromatase inhibitor
RCT: Randomised controlled trial
ISRCTN: International Standard Randomised Controlled Trial Number
ACT: Acceptance and commitment therapy
NHS: National Health Service
UK: United Kingdom
HCPC: Health and Care Professional Council
UKCP: UK Council for Psychotherapy
TNM: Tumour, Node, Metastasis
AJCC: American Joint Committee on Cancer
CTRU: Clinical Trials Research Unit
TIDieR: Template for Intervention Description and Replication
DOSE: Domains of Subjective Extent
EQ-5D-5L: EuroQol-5 dimension 5-level
MQoL-R: McGill Quality of Life-Revised
SEAMS: Self-Efficacy for Appropriate Medication Use Scale
SRBAI: Self-Report Behavioural Automaticity Index
BMQ-AET: Beliefs about Medicines Questionnaire- Adjuvant Endocrine Therapy
MPFI: Multi-Dimensional Psychological Flexibility Index
DASS-21: Depression Anxiety Stress Scales
EORTC: European Organisation for Research and Treatment of Cancer
UKCC: UK Cancer Costs Questionnaire
SWAT: Study Within a Trial
GP: General Practitioner
REDCap: Research Electronic Data Capture
ACT-FM: Acceptance and Commitment Therapy- Fidelity Measure
LIHS: Leeds Institute of Health Sciences
TMG: Trial Management Group
TSC: Trial Steering Committee
RUSAE: Related or unexpected serious adverse event
REC: Research Ethics Committee
SAE: Serious adverse event
