# Supplementary material for: Optimising a behavioural intervention to support endocrine therapy adherence for women with breast cancer: protocol for the ROSETA optimisation factorial randomised controlled trial
Source: Trials. 2026 May 11;27:463. doi: 10.1186/s13063-026-09765-6 (PMC13330054; doi:10.1186/s13063-026-09765-6)
Supplement: Supplementary file 1 — Additional file 1. Objectives and Endpoints: detailed objectives with linked endpoints. [file 13063_2026_9765_MOESM1_ESM.pdf]

Additional File 1: Objectives and Endpoints

Primary Objective and Endpoint

**Objective**	**Endpoint**
To determine the most effective intervention package for supporting AET adherence at 12months post-randomisation	Estimate the main effects and all interactions of four intervention components measured using the DOSENonadherence at 12-months post-randomisation

Secondary Statistical Objectives and Endpoints

**Objective**<br>*To determine the most effective intervention package for:*	**Endpoint**<br>*Estimate the main effects and all interactions of four intervention components measured using:*
Supporting AET adherence and persistence at 12-months post-randomisation	NHS prescribing and/or dispensing data at 12-months post-randomisation
Global quality of life at 12-months postrandomisation	EQ-5D-5L and MQoL-R scales at 12-months postrandomisation
Self-efficacy at 12-months postrandomisation	SEAMS at 12-months post-randomisation
Supporting AET adherence at 12-months post-randomisation considering key restraints such as cost	DOSE-Nonadherence at 12-months post-randomisation
**Objective**<br>*To estimate the main effects and interactions of the intervention components for:*	**Endpoint**<br>*Estimate the main effects and all interactions of four intervention components measured using:*
Supporting AET adherence at 4- and 8months post-randomisation	DOSE-Nonadherence at 4- and 8-months postrandomisation
Global quality of life at 4- and 8-months postrandomisation	EQ-5D-5L and MQoL-R scales at 4- and 8-months postrandomisation
Self-efficacy at 4- and 8-months postrandomisation	SEAMS at 4- and 8--months post-randomisation
Component specific	
To estimate the effect of the SMS component on habit formation at 4-, 8- and 12-months post-randomisation	SRBAI at 4-, 8- and 12-months post-randomisation
To estimate the effect of the information leaflet component on beliefs about medication at 4-, 8- and 12-months post-randomisation	BMQ-AET at 4-, 8- and 12-months post-randomisation

To estimate the effect of the ACT component on psychological flexibility at 4-, 8- and 12months post-randomisation	MPFI at 4-, 8- and 12-months post-randomisation
To estimate the effect of the ACT component on psychological distress at 4-, 8- and 12months post-randomisation	DASS-21 at 4-, 8- and 12-months post-randomisation
To estimate the effect of the website component on symptomatic quality of life at 4, 8- and 12-months post-randomisation	EORTC QLQ-C30, EORTC QLQ-BR45, EORTC-IL133 at 4-, 8- and 12-months post-randomisation

Secondary Health Economic Objectives and Endpoints

Objective	Endpoint
To estimate the cost of developing and delivering each intervention component at 12-months post-randomisation	NHS reference costs, PSSRU cost data and UK Cancer Costs questionnaire at 12-months post-randomisation

Process Evaluation Objectives and Endpoints

Objective	Endpoint
Empirically test the conceptual model by estimating the causal pathways through which the intervention components are intended to operate	DOSE-Nonadherence, EQ-5D-5L, EORTC QLQ-C30, EORTC QLQ-BR45, EORTC-IL133, MPFI, BMQ-AET, SRBAI, DASS-21, MQoL-R, SEAMS, NHS England prescribing and dispensing data
Identify i) whether there are latent trajectories of the mediating variables, engagement with ACT skills and use of the website; ii) the factors associated with class membership; and iii) the association between latent trajectories, medication adherence, global QoL and self-efficacy	DOSE-Nonadherence, EQ-5D-5L, EORTC QLQ-C30, EORTC QLQ-BR45, EORTC-IL133, MPFI, BMQ-AET, SRBAI, DASS-21, MQoL-R, SEAMS, NHS England prescribing and dispensing data, engagement with ACT skills, website usage data
Undertake a longitudinal qualitative participant interview study to i) explore the mechanisms through which the intervention components may be working and ii) explore the fidelity of receipt and enactment of the intervention components	Qualitative interviews with trial participants
To assess the acceptability and fidelity of the intervention components with regard to training, delivery, receipt and enactment	Qualitative interviews with trial participants and therapists<br>ACT-Fidelity Measure (ACT-FM) competency scores as assessed via role play, for the first 5 therapy tapes and via an external reviewer<br>Treatment data (SMS delivery and receipt, website and information leaflet delivery, website usage, ACT procedural fidelity checklists, ACT attendance and engagement)<br>Intervention adherence, at 4-months post-randomisation

	Use of ACT skills, at 4-, 8- and 12-months post-randomisation
To triangulate the process evaluation findings to refine the conceptual model	No endpoints defined for this objective
