# Supplementary material for: Optimising a behavioural intervention to support endocrine therapy adherence for women with breast cancer: protocol for the ROSETA optimisation factorial randomised controlled trial
Source: Trials. 2026 May 11;27:463. doi: 10.1186/s13063-026-09765-6 (PMC13330054; doi:10.1186/s13063-026-09765-6)
Supplement: Supplementary file 2 — Additional file 2. SPIRIT Factorial trial extension checklist. [file 13063_2026_9765_MOESM2_ESM.pdf]

Section/ topic	Item No.	SPIRIT 2013 Checklist item	Extension for factorial trials	Page number
Title	1	Descriptive title identifying the study design, population, interventions, and, if applicable, trial acronym	Descriptive title identifying the study as a factorial randomized trial, as well as the population, interventions, and, if applicable, trial acronym	1
Background and rationale	6a	Description of research question and justification for undertaking the trial, including summary of relevant studies (published and unpublished) examining benefits and harms for each intervention	Description of research question and justification for undertaking the trial, including summary of relevant studies (published and unpublished) examining benefits and harms for each intervention, and rationale for using a factorial design, including whether an interaction is hypothesized	5-6
Objectives	7	Specific objectives or hypotheses	Specific objectives or hypotheses and a statement of which treatment groups form the main comparisons	6
Trial design	8	Description of trial design including type of trial (eg, parallel group, crossover, factorial, single group), allocation ratio, and framework(eg, superiority, equivalence, noninferiority, exploratory)	Description of the type of factorial trial (eg, full or partial, number of factors, levels within each factor), allocation ratio, and framework (eg, superiority, equivalence, noninferiority, exploratory)	7
Eligibility criteria	10	Inclusion and exclusion criteria for participants. If applicable, eligibility criteria for study centers and individuals who will perform the interventions (eg, surgeons, psychotherapists)	Inclusion and exclusion criteria for each factor, noting any differences if applicable. If applicable, eligibility criteria for study centers and individuals who will perform the interventions (eg, surgeons, psychotherapists)	8
Sample size	14	Estimated number of participants needed to achieve study objectives and how it was determined, including clinical and statistical assumptions supporting any sample size calculations	Estimated number of participants needed to achieve study objectives and how it was determined for each main comparison, including clinical and statistical assumptions supporting any sample size calculations, such as whether an interaction was assumed in the calculation	14
Sequence generation	16a	Method of generating the allocation sequence (eg, computer-generated	Method of generating the allocation sequence (eg, computer-generated random numbers), list of any variables for stratification, and	15

		random numbers), and list of any factors for stratification. To reduce predictability of a random sequence, details of any planned restriction (eg, blocking) should be provided in a separate document that is unavailable to those who enroll participants or assign interventions	whether participants were allocated to factors at different time points, if applicable. To reduce predictability of a random sequence, details of any planned restriction (eg, blocking) should be provided in a separated document that is unavailable to those who enroll participants or assign interventions	
Statistical methods	20a	Statistical methods for analyzing primary and secondary outcomes. Reference to where other details of the statistical analysis plan can be found, if not in the protocol	Statistical methods for each main comparison for primary and secondary outcomes, including:<br>• Whether the target treatment effect for each main comparison pertains to the effect in the presence or absence of other factors;<br>• Approach, such as factorial or multi arm;<br>• How the approach will be chosen, such as pre-specified or based on estimated interaction;<br>• If factorial approach to analysis will be used, whether factors will be adjusted for each other;<br>• Method(s) for evaluating statistical interactions, and which outcomes (in addition to the primary) they will be applied to;<br>• If applicable, how non-concurrent recruitment to factors will be handled;<br>• Reference to where other details of the statistical analysisplan can be found, if not in the protocol	20-21
Data monitoring	21b	Description of any interim analyses and stopping guidelines, including who will have access to these interim results and make the final decision to terminate the trial	Description of any interim analyses and stopping guidelines, noting any differences across main comparisons, with reasons, and who will have access to these interim results and make the final decision to terminate the trial	22
