# Supplementary material for: Optimising a behavioural intervention to support endocrine therapy adherence for women with breast cancer: protocol for the ROSETA optimisation factorial randomised controlled trial
Source: Trials. 2026 May 11;27:463. doi: 10.1186/s13063-026-09765-6 (PMC13330054; doi:10.1186/s13063-026-09765-6)
Supplement: Supplementary file 3 — Additional file 3. TIDieR checklist of intervention components. [file 13063_2026_9765_MOESM3_ESM.pdf]

Additional File 2: TIDieR Checklist

Nº	What	Details
**1**	**Name**	Optimising a behavioural intervention to support endocrine therapy adherence for women with breast cancer: Protocol for the ROSETA optimisation randomised controlled trial
**2**	**Why: Rationale, theory, goal**	Adjuvant endocrine therapies are prescribed at the end of hospital-based breast cancer treatment to prevent recurrences and all-cause mortality. However, adherence to these medications is often poor, due to multiple factors, including forgetting, beliefs about medications, intolerable side-effects and psychological distress. This time during the cancer journey is also particularly challenging, as women are transitioning from 'patient' to 'survivor'. They also report a lack of support during this time, post hospital discharge.<br><br>Previous adherence interventions have tended to consist of solely educational based interventions that are not grounded in theory, and did not target the factors commonly associated with medication adherence. Given the wide range of barriers to adherence in this population, it is perhaps unsurprising that previous interventions have shown limited effectiveness. An alternative strategy is to design a complex intervention, with multiple components that can target a range of factors that have been highlighted as barriers to adherence.<br><br>*Memory and forgetting*<br>Mobile phone-based interventions are well suited to tackle forgetfulness as a barrier to adherence, through reminders and promotion of habit formation. SMS messages have been shown to be effective in improving medication adherence in other chronic illnesses but have not been widely tested in cancer patients.<br><br>*Medication schemas*<br>Accurate information about the necessity and risks of AET has the potential to increase women's perceptions of their need for AET, and to reduce unfounded concerns about the medication. In addition, women with breast cancer have stated that they would like more accurate information about AET to overcome unfounded concerns.<br><br>*Psychological Flexibility*<br>ACT has been shown to improve outcomes in those living with chronic illness, chronic pain, and cancer. ACT aims to increase a participant's awareness of their personal values, and to undertake more of the behaviours that support these values – a process that often involves developing a willingness to have painful thoughts and feelings (such as

		medication side-effects). ACT targets psychological flexibility, which can improve functioning during objectively difficult circumstances, and can often reduce psychological distress as a by-product.<br><br>*Living with Side-effects*<br><br>One of the most commonly cited barriers to AET adherence is the impact of side-effects, and the lack of support for management of these is commonly cited. There are a number of strategies for these side-effects that have the potential to be effective in alleviating symptoms. However, these are typically not presented in a patient-friendly manner.<br><br>Given the above, we have co-designed four intervention components for women with breast cancer who have been prescribed adjuvant endocrine therapies; SMS reminder messages to target forgetfulness, an information leaflet to promote formation of accurate beliefs, ACT therapy sessions to increase psychological flexibility, and a side-effect management website to support living with side-effects. The aim of the intervention components are to support medication adherence to endocrine therapy. Participants will be randomised to receive none, or a combination of one or more of these four components.
**3**	**What Materials**	***Participants randomised to receive SMS component***<br>Participants will receive 43 SMS messages over four months. This includes three opening messages, one closing message, 36 messages related to BCTs aiming to promote habit formation, and 3 messages (sent after 1, 2 and 3 months) as a reminder that participants can stop any further SMS messages being sent by emailing the ROSETA team. The content of the SMS messages was co-developed with experts in behaviour change and/or medication adherence, and women who have experienced breast cancer.<br><br>***Participants randomised to receive information leaflet***<br>Participants will receive an information leaflet containing detailed information about AET. This includes information about how the medication works (with diagrams to supplement), information about the benefits and side-effects of AET, answers to common concerns that women have, and quotes from women with experience of taking AET.<br><br>***ACT***<br>***Participants randomised to ACT sessions***

		Participants will be emailed a participant manual consisting of information about the ACT skill and home practice tasks, in addition to corresponding audio files to assist with the home practice tasks. Each of the four modules focuses on a different ACT-based skill:<br>• Module 1: Mindfulness and unhooking<br>• Module 2: Following your values<br>• Module 3: Taking an observer perspective<br>• Module 4: Recap, reflection, and staying committed<br>***Therapists delivering ACT sessions***<br>Therapists delivering the intervention will receive two or three half days (see no. 5 below) of bespoke training delivered by clinical psychologists with ACT experience. Alongside this, they will receive a training manual, with information about ACT generally, and specific session plans for the intervention sessions.<br>***Participants randomised to receive access to side-effect website***<br>Participants allocated to receive the website will receive access to a bespoke website containing information and strategies for self-management of side-effects, and signposting to further sources of support.
**4**	**What Procedures**	***Intervention Delivery***<br>***Participants randomised to receive SMS component***<br>Participants will receive 43 SMS messages over four months. These include three opening messages, one closing message, 36 messages related to BCTs aiming to promote habit formation, and 3 messages (sent after 1, 2 and 3 months) as a reminder that participants can stop any further SMS messages being sent by emailing the ROSETA team. The content of the SMS messages was co-developed with experts in behaviour change and/or medication adherence, and women who have experienced breast cancer.<br>The 36 messages relating to BCTs will be sent on the following schedule:<br>- Daily messages for 2 weeks<br>- Two messages per week for 8 weeks<br>- Weekly messages for 6 weeks

		Participants randomised to receive information leaflet<br>Participants will be sent the information leaflet by email 1 week post-randomisation.<br><br>***Participants randomised to receive ACT***<br>4x guided self-help modules consisting of information about ACT, home practice exercises and corresponding audio files<br>1x 25-minute individual introductory session with a trial therapist<br>3 x 25 minute individual support sessions with a trial therapist to discuss the module completed over the past week, their experiences of the home practice exercises, and to allow discussion of any difficulties that arose.<br>1x 25-minute closing session with a trial therapist<br><br>***Participants randomised to receive access to side-effect website***<br>Participants will be given login details of the website 1 week post-randomisation.<br><br>***Therapist Training***<br>(See no. 5, below, for information on this)<br><br>***Evaluation of the Intervention Components***<br>Adherence, quality of life/symptom burden, self-efficacy, psychological distress, psychological flexibility, habitual behaviour of medication taking and medication beliefs will be measured at baseline, and 4-, 8- and 12-months post-randomisation. Self-reported receipt and engagement of intervention components will be monitored. SMS delivery, and website use will be tracked.<br><br>***Support activities***<br>Recruitment and consent of participants
**5**	**Who provided**	***SMS messages***<br>The CTRU will send all SMS messages to participants.<br><br>***Information Leaflet***<br>The information leaflet will be sent to participants by the site.<br><br>***ACT***

		The therapists who will deliver the intervention will undergo training in delivering ACT. Training delivery will be tailored according to current role and grade. Assistant psychologists, clinical associate psychologists and psychological wellbeing practitioners (or equivalent) will be required to complete 3 half days of training. HCPC registered practitioner psychologists and UKCP registered psychotherapists will be required to complete two of the half day sessions. The training will be delivered by a clinician with expertise in ACT applied to chronic disease. Training includes teaching about ACT and practice of intervention-specific therapy methods.<br><br>Each site's therapists may have a varied background that may or may not have included previous ACT training prior to our delivered training programme. Eligible therapists included:<br>• HCPC registered practitioner psychologist (Clinical, Health or Counselling Psychologist)<br>• UKCP registered psychotherapist<br>• Assistant psychologist<br>• Clinical associate psychologist<br>• Individual with formal experience or training in delivering manualised psychological therapy for supporting wellbeing (e.g., a psychological wellbeing practitioner)<br><br>***Website***<br>Access to the bespoke website will be provided by the site.
**6**	**How: mechanisms of delivery**	***Participants randomised to receive SMS component***<br>SMS messages will be sent in an automated fashion by the CTRU to the participants mobile phone based on the following schedule:<br><br>- Daily messages for 2 weeks<br>- Two messages per week for 8 weeks<br>- Weekly messages for 6 weeks<br>In addition one message will be sent after months 1, 2 and 3 as a reminder that participants can stop any further SMS messages being sent by emailing the ROSETA team.<br><br>***Participants randomised to receive information leaflet***<br>Participants will be sent the information leaflet electronically 1 week post-randomisation and will be able to read this as they wished.<br><br>***Participants randomised to receive ACT***

		The individual sessions (5 in total) will be delivered via phone or video conferencing.<br><br>The participant manual containing information about each module, home practice tasks, and audio files will be emailed to each participant by the therapist following each session.<br><br>***Participants randomised to receive access to side-effect website***<br>Participants will be given a login to the website 1 week post-randomisation and will be able to use this as they wished.
**7**	**Where: location of delivery**	***SMS Messages***<br>Not applicable.<br><br>***Information Leaflet***<br>Not applicable.<br><br>***ACT***<br>All sessions will be delivered remotely via phone or videoconferencing.<br><br>***Website***<br>Not applicable
**8**	**When and how much**	***Participants randomised to receive SMS component***<br>SMS messages will be sent by the CTRU based on the following schedule:<br><br>- Daily messages for 2 weeks<br>- Two messages per week for 8 weeks<br>- Weekly messages for 6 weeks<br><br>In addition three opening messages, one closing message and, one message after months 1,2 and 3 will be sent reminding participants that they could stop any further SMS messages being sent via emailing the ROSETA team.<br><br>***Participants randomised to receive information leaflet***<br>Participants will be sent the information leaflet electronically 1 week post-randomisation and will be able to read this as they wished.<br><br>***Participants randomised to receive ACT***

		All support sessions will last up to 25 minutes. Participants will be added to an ACT waiting list at their respective site with sessions beginning as soon as a therapist is available. The therapy sessions were recommended to be held fortnightly, with all sessions being completed within 3 months of the participant's first ACT session.<br><br>***Participants randomised to receive access to side-effect website***<br>Participants will be given a login to the website 1 week post-randomisation and will be able to use this as they wished.
**9**	**Tailoring**	***SMS***<br>The same SMS messages will be sent in the same order to each participant. Participants will be able to select the time of day they wish the messages to be sent; options are morning, lunch and evening.<br><br>***Information Leaflet***<br>The same information leaflet will be sent to each participant.<br><br>***ACT***<br>Although there is a set session plan to follow, detailing specific exercises and tasks for each session, the therapy itself is quite flexible. As such, the deliverer may adapt the content to ensure it's relevant to each participant (e.g. through discussing specific individuals' values, goals, and behaviours).<br><br>***Website***<br>The website will be the same for each participant.
**10***	**Modifications**	*<To be completed post study completion>*
**11**	**How well (planned)**	***SMS***<br>Successful delivery and receipt of the SMS messages will be recorded by the CTRU, alongside the number of SMS messages that were unable to be delivered. Participants will answer a single item asking whether they received the SMS messages, and another item asking how many of the SMS messages they read. Semi-structured interviews will be conducted to understand fidelity of receipt and enactment of the messages.<br><br>***Information Leaflet***<br>The number of information leaflets sent out to participants will be recorded. This will be recorded by the site when each information leaflet is sent out.

		Participants will be asked to self-report whether they received the information leaflet, and how much of the information leaflet they read. Semi-structured interviews will be conducted with participants to understand the fidelity of receipt and enactment of the information leaflet.<br><br>***ACT***<br>At the end of the training and prior to delivery of ACT to trial participants, trial therapists will be required to undertake a role play reflecting a clinical scenario with the ACT intervention training lead or delegate to demonstrate their competency in delivering the ACT component. The ACT-FM therapist stance subscale will be used to assess competency. After competency role plays have been conducted with 10 therapists who are HCPC registered practitioner psychologists/UKCP registered physiotherapists, we will review the need for the competency assessment. If it is clear that the training is sufficient for this level of therapist then this competency assessment will be removed. The competency assessment will remain throughout the trial for the training of assistant psychologists, clinical associate psychologists and psychological wellbeing practitioners (or equivalent).<br><br>A further competency assessment will monitor competency of assistant psychologists, clinical associate psychologists and psychological wellbeing practitioners (or equivalent). The first 5 therapy tapes from the trial (maximum 1 per therapist) will be assessed by the ACT intervention training lead or delegate who will assess the sessions based on the ACT-FM. If 3 or more are considered competent then training will be assumed to be sufficient. If competency is not met in three or more of the five tapes reviewed, then appropriate changes will be made to the training specific to the assistant psychologists clinical associate psychologists and psychological wellbeing practitioners (or equivalent). As a further check for competency of assistant psychologists, clinical associate psychologists or psychological wellbeing practitioners (or equivalent), the ACT intervention training lead or delegate will review the first five therapy tapes in the trial from session 2, 3 or 4 with participants who have consented to be audio recorded. A maximum of one session for each trial therapist will be assessed for competency. ACT intervention training lead or delegate will rate the selected sessions using the ACT-FM therapist stance subscale. To be considered competent in ACT, trial therapists will need to score >4 on the ACT consistent subscale, and <5 on the ACT inconsistent subscale. If the majority of tapes reach competency (3 or more), then it will be assumed that training is sufficient and no changes will be made. If competency is not met in three or more of the five tapes reviewed, then appropriate changes will be made to the training specific to the assistant psychologists clinical associate

		psychologists and psychological wellbeing practitioners (or equivalent). Competency is assumed for higher band therapists following the results from the ROSETA pilot.<br><br>Therapist fidelity to competently deliver the intervention in line with ACT will be further assessed by an external rater with a background in ACT. They will complete the ACT-FM therapist stance subscale checklist whilst listening to the audio recording of 20 sessions. A score of >4 on ACT consistent behaviours and <5 on ACT inconsistent behaviours is considered competent.<br><br>Additionally, an intervention specific metric of "Procedural Fidelity" is included, which measures other aspects of the intervention that are important for treatment fidelity but are not ACT-specific (e.g. reflecting on home practice tasks, sending module content etc). Therapists will complete the procedural fidelity checklist following each session. A percentage score is created for each session by dividing the score achieved by the maximum possible score achievable within that session and multiplying by 100.<br><br>Participant fidelity to the ACT component will be monitored by recording the number of sessions attended, missed and cancelled. The therapist additionally reported how much of the module materials the participant had read and engaged with (participant manual, audio files and home practice tasks). Participants self-reported receipt of the module content, self-reported engagement with the participant manual, audio files and home practice tasks. Semi-structured interviews additionally assessed fidelity of receipt and enactment.<br><br>***Website***<br>Website data will be tracked for each participant, including number of logins, time spent on pages, videos watched and clicked links. Participants will be asked a single item about their engagement with the website. Fidelity of receipt and enactment will be additionally assessed through semi-structured interviews.
**12***	**How well (actual)**	*<To be completed post study>*
